# Complete mitochondrial genome sequence of *Dolichovespula kuami* (Hymenoptera: Vespidae)

**DOI:** 10.1080/23802359.2026.2620190

**Published:** 2026-02-01

**Authors:** Byung Kwon Pi, Moon Bo Choi, Young-Ho Ha, Chang-Jun Kim, Seung-Su Euo

**Affiliations:** a Division of Forest Biodiversity, Korea National Arboretum, Pocheon, Republic of Korea; b Institute of Agricultural Science and Technology, Kyungpook National University, Daegu, Republic of Korea; c Department of Forest Bio-resources, National Institute of Forest Science, Suwon, Republic of Korea

**Keywords:** Insecta, Vespidae, mitochondrial genome, *Dolichovespula kuami*

## Abstract

This study is part of an ongoing systematic research on the Korean Vespidae fauna. The complete mitochondrial genome of the social wasp *D. kuami* collected in South Korea was sequenced and annotated. The circular genome was 15,671 bp long and comprised 13 protein-coding genes, 22 transfer RNA genes, and two ribosomal RNA genes. Phylogenetic analysis revealed that *D. kuami* formed a monophyletic clade with other *Dolichovespula* species, indicating its distinctiveness despite being the sister group to *D. flora*. These findings confirm the taxonomic status of *D. kuami* and provide valuable genomic data for future phylogenetic studies of the Vespidae family.

## Introduction

Social wasps form hierarchical societies composed of queens, workers, and males (Hines et al. 2007; Wyatt et al. 2023). These insects cooperate in nest building, with queens reproducing, and workers foraging, caring for the brood, and defending the nest (Toth and Rehan 2017; Wyatt et al. 2023). This highly organized social structure represents a major evolutionary transition from solitary lifestyles, enabling more efficient resource use and greater reproductive success (Toth and Rehan 2017).

Among these social wasps, the genus *Dolichovespula* is notable for its ecological role and species diversity. Approximately 20 species are currently recognized worldwide (Archer 2006; Carpenter et al. 2011; Kim et al. 2024), with four reported in South Korea (KSAE and ESK 2021). In addition to their role as predators, certain species, such as *D. sylvestris* (Scopoli 1763), act as pollinators, indicating broader ecological roles (Proctor et al. 1996).

Despite their ecological significance, *Dolichovespula* species often exhibit morphological similarities, complicating species-level identification and resulting in taxonomic ambiguities (Archer 1999). To overcome these limitations, molecular phylogenetic approaches have been widely adopted. Specifically, DNA barcoding using the cytochrome c oxidase subunit I (*COX1*) gene has proven to be a straightforward and effective method for insect taxonomy (Hebert et al. 2003a, 2003b).

*Dolichovespula kuami* Kim and Yoon (1996), was first described in South Korea as a novel species. Belonging to the *D. maculata* (Linnaeus 1763) species group, it closely resembles *D. flora* Archer 1987, a member of the same group that was previously considered to be conspecific (Archer 1999, 2006; Tan et al. 2014). However, Kim et al. (2024) proposed its independent species status based on clear molecular and ecological differences, including variations in pronotal striation, male genitalia, and *COX1* barcode sequences.

This study aimed to decode the complete mitochondrial genome of *D. kuami* to reassess and support the claims made by Kim et al. (2024). Using genomic evidence, we sought to establish the taxonomic position of *D. kuami*, thereby contributing to a deeper understanding of species boundaries within the genus *Dolichovespula*.

## Materials and methods

**Figure 1.**
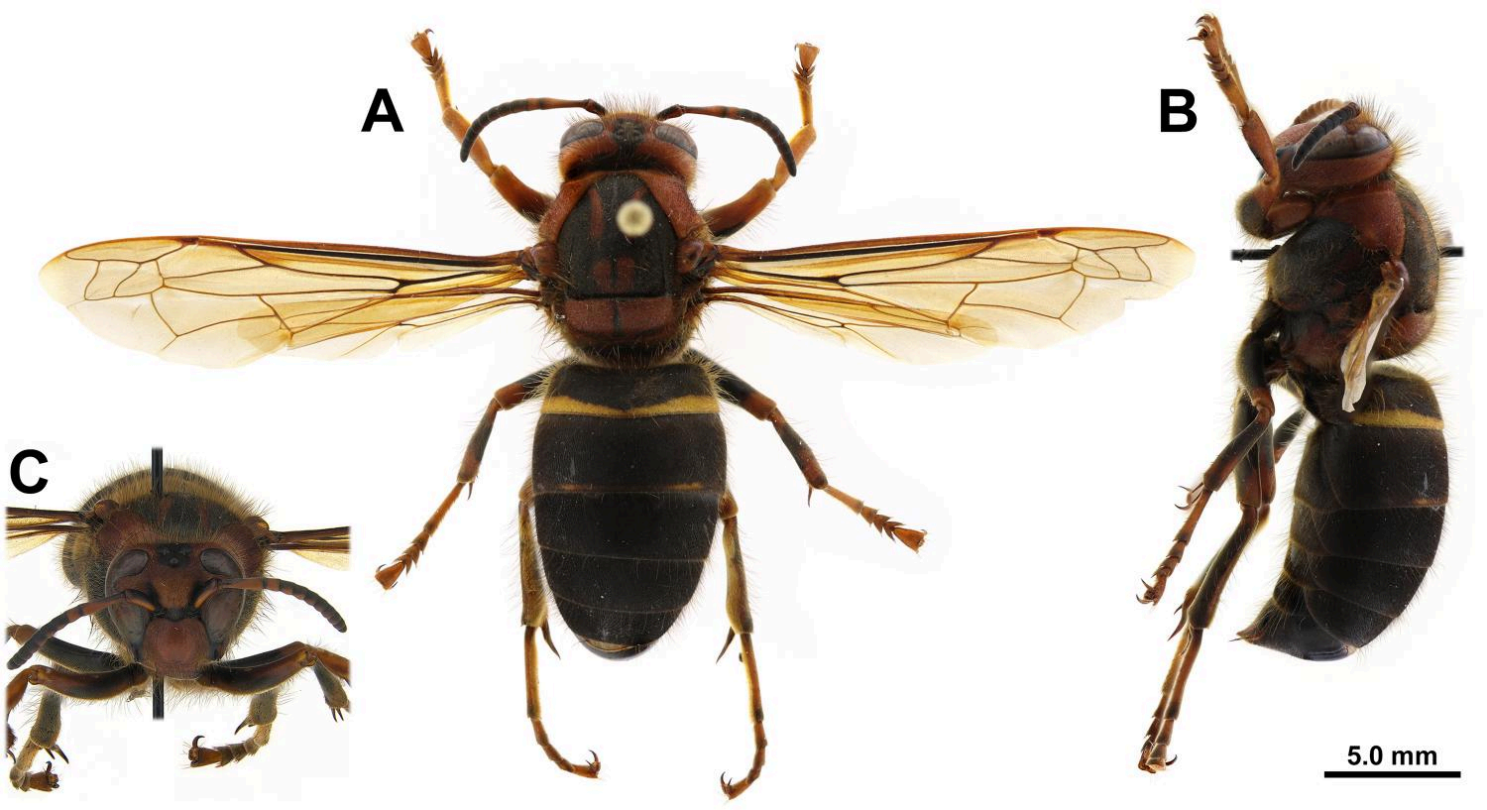
*Dolichovespula kuami* female. A. Habitus, dorsal view; B. Habitus, lateral view; C. Head, frontal view (photographed by Seung-Su Euo).

With permission from the Korea Forest Service, a specimen (Figure 1) was collected using a Malaise trap at Haksodaepokpo, Mt. Unmunsan, Gyeongsangbuk-do, South Korea (N35˚38′15″ E128˚59′51″), between June 22 and 28, 2014. It was identified as a female *Dolichovespula kuami* based on the following morphological characteristics—dull and indistinct pronotal rugae, faint longitudinal rugae on the lateral surface of the pronotum near the pronotal pit, fine vertical rugae on the posterior portion, and the distances between the posterior ocelli and between the anterior and posterior ocelli were nearly equal to the diameter of the anterior ocellus (Kim 2011; Kim et al. 2024). A voucher specimen (KNAE468966) was deposited at the Korea National Arboretum (https://kna.forest.go.kr; Chang-Jun Kim, changjunkim@korea.kr).

Digital images of different focal planes were captured using a Dhyana 400DC camera (Tucsen Photonics, China) mounted on a Leica DM3000 LED microscope (Leica Microsystems, Germany) and processed using Helicon Focus v8.3.0 (Helicon Soft, Ukraine).

**Figure 2.**
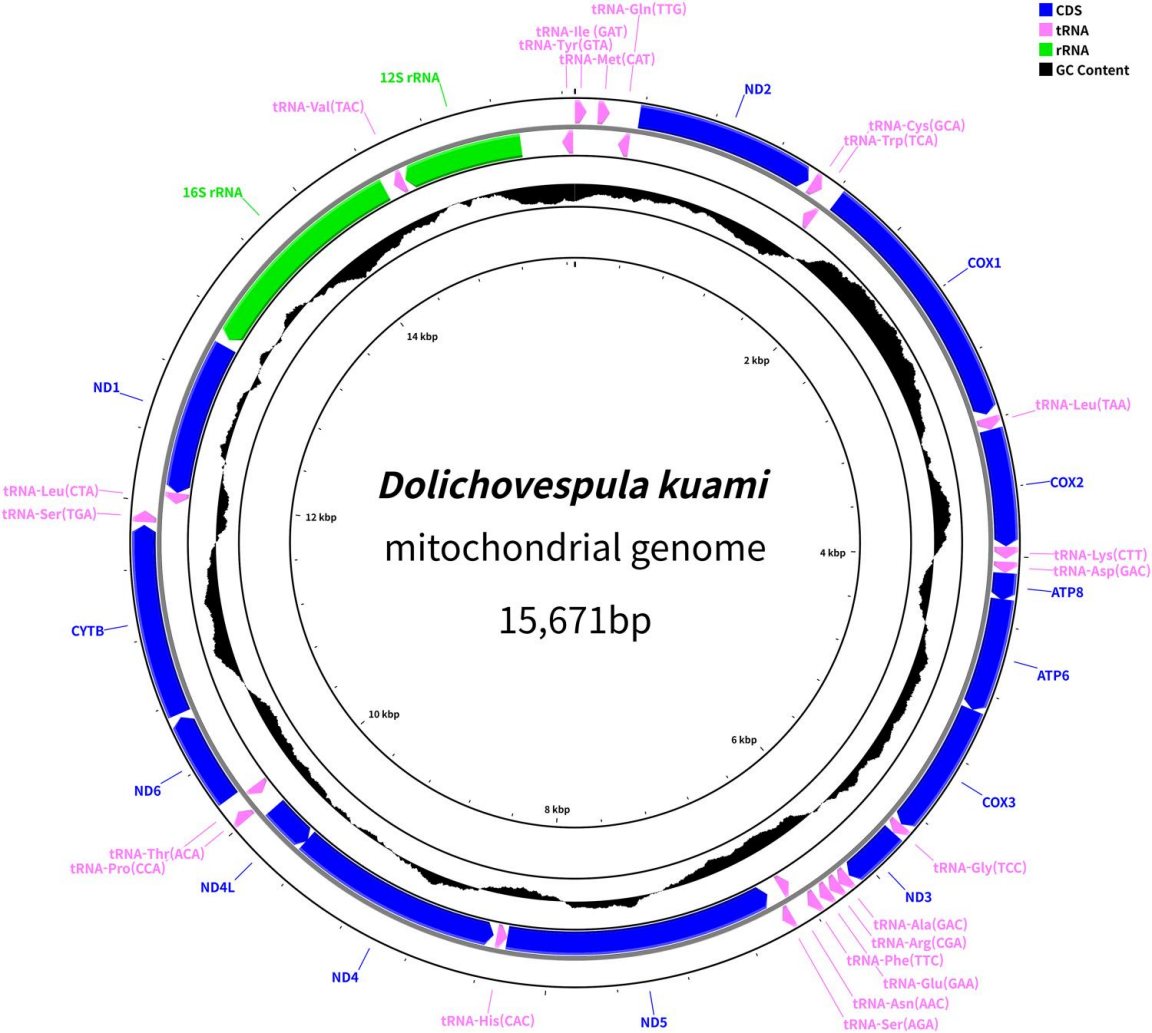
Circular map of the mitochondrial genome of *Dolichovespula kuami*. The complete mitochondrial genome is 15,671 bp in length and is illustrated with a circular layout. Coding DNA sequence (CDS), tRNAs, and rRNAs are color-coded according to the legend. The innermost plot represents the GC content across the genome, with black bars indicating the degree of deviation from the average GC content.

Genomic DNA was extracted from the legs using a DNeasy Blood & Tissue Kit (Qiagen, Germany). Sequencing libraries were prepared using the TruSeq Nano DNA Sample Preparation Kit (Illumina Inc., USA) following the manufacturer’s protocol. Sequencing on the Illumina HiSeq4000 platform (Macrogen, Inc., South Korea) generated 24,156,248 reads (3,647,593,448 bp), of which 18,327,422 reads (2,759,212,228 bp) were retained after quality filtering with FastQC. *De novo* assembly was performed using SOAPdenovo (Li et al. 2010) with default parameters. BLAST analysis (*E*-value < 1e–3) identified 26,732 mitochondrial reads (4,012,658 bp), which were reassembled with the same program to produce 21 contigs totaling 24,354 bp. The terminal regions were extended and merged in Geneious Prime 2022.2.2 (Kearse et al. 2012), yielding a complete 15,671 bp circular genome. Circularity was verified by mapping all raw reads to the assembled sequence using the “Map to Reference” function in Geneious, which showed uniform coverage without gaps or indels. Mapping statistics were evaluated with QualiMap v2.3 (Okonechnikov et al. 2016), and coverage visualized in R (ggplot2) (Figure S1). Gene annotation and circular mapping were performed using MITOS2 (Bernt et al. 2013) and Proksee (Grant et al. 2023) (Figure 2).

For phylogenetic analysis, 29 complete wasp mitogenomes showing over 69% identity with *D. kuami* (NC_069159.1) were retrieved from GenBank (April 2025) using BLASTn (Camacho et al. 2009) (Table S1). Only concatenated protein-coding genes (PCGs) and ribosomal RNA (rRNA) sequences, excluding transfer RNA (tRNA) genes, were used for phylogenetic analyses. Polistinae species served as outgroups (Wang et al. 2022). Alignments were performed in Geneious Prime 2022.2.2 with Clustal Omega 1.2.2 (Sievers et al. 2011). Maximum-likelihood analysis was conducted in IQ-TREE v3.0.1 (Nguyen et al. 2015) with 10,000 ultrafast bootstrap replicates (UFBoot2; Hoang et al. 2018). The best-fit model (GTR+F + R4) was selected by ModelFinder under the Bayesian Information Criterion (BIC).

## Results

**Figure 3.**
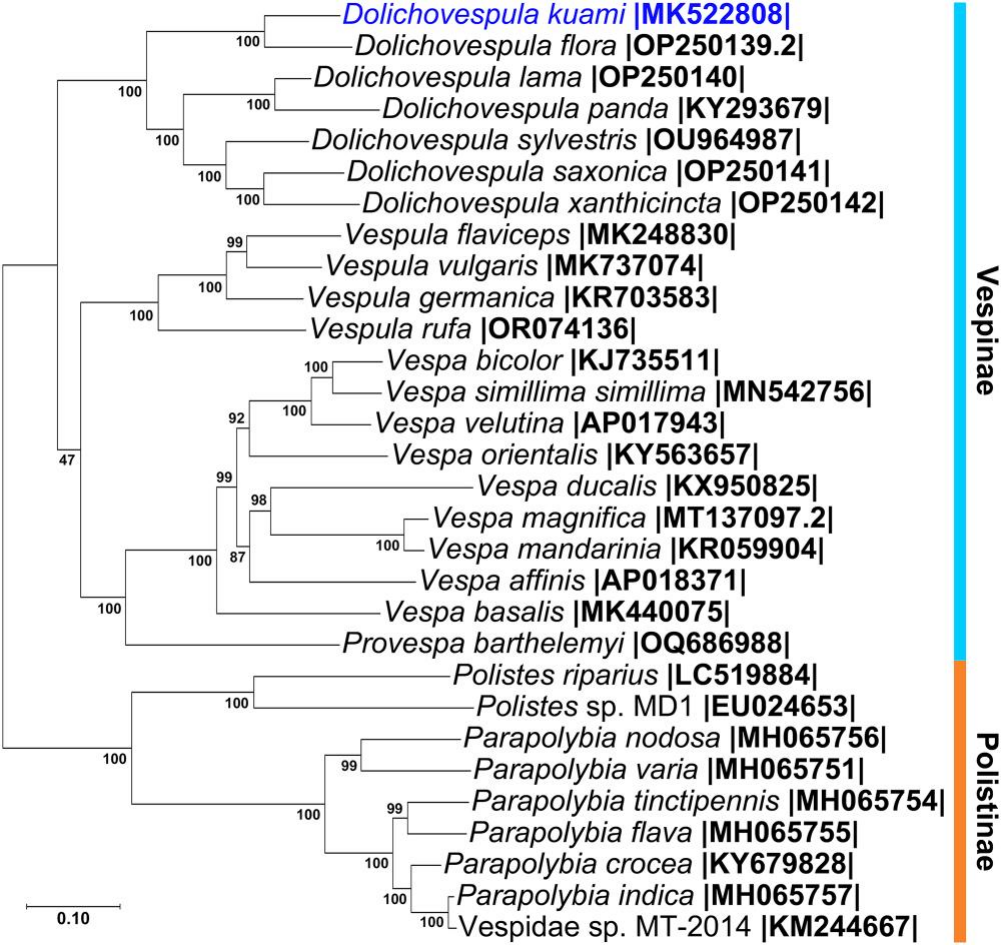
Maximum-likelihood phylogenetic tree based on complete mitochondrial genomes of 30 vespid wasps. The tree is constructed using the newly sequenced mitochondrial genome of *Dolichovespula kuami* (MK522808, this study) along with 29 additional sequences obtained from the NCBI GenBank database (as of April 2025). The following sequences were used: OP250139.2 (Wang et al. 2022), OP250140 (Wang et al. 2022), KY293679 (Fan et al. 2017), OU964987 (Falk and Broad 2022), OP250141 (Wang et al. 2022), OP250142 (Wang et al. 2022), MK248830 (Zhao et al. 2019b), MK737074 (Dobelmann et al. 2019), KR703583 (Song et al. 2016), OR074136 (Euo et al. 2024), KJ735511 (Wei et al. 2016), MN542756 (Choi et al. 2019), AP017943 (Takahashi et al. 2017), KX950825 (Kim et al. 2017), MT137097.2 (Feng et al. 2022), KR059904 (Chen et al. 2016), AP018371 (Okuyama et al. 2017), KY563657 (Haddad et al. 2017), MK440075 (Zhao et al. 2019a), OQ686988 (Liu et al. 2023), LC519884 (Yamasaki et al. 2020), EU024653 (Cameron et al. 2008), MH065756 (Luo et al. 2022), MH065751 (Luo et al. 2022), MH065754 (Luo et al. 2022), MH065755 (Luo et al. 2022), KY679828 (Peng et al. 2017), MH065757 (Luo et al. 2022), KM244667 (Tang et al. 2014). Bootstrap support values are indicated at each node.

The complete mitochondrial genome of *D. kuami* is 15,671 base pairs long and contains 13 PCGs, 22 tRNA genes, and two rRNA genes (Figure 2). Among these, nine PCGs and 14 tRNA genes were transcribed from the forward strand, whereas four PCGs, eight tRNAs, and both rRNAs were encoded on the reverse strand. Most PCGs used the standard ATG start codon, while a few employed alternative codons (ATC, ATT, or ATA). All but one PCG terminated with TAA, whereas NAD5 ended with TAG (see Table S2). The mitochondrial genome has a nucleotide composition of 41.8% adenine, 40.2% thymine, 6.0% guanine, and 12.0% cytosine, with a GC content of 18.0%. In the resulting phylogenetic tree (Figure 3), *D. kuami* clustered with other *Dolichovespula* species within the subfamily Vespinae. It formed a sister group with *D. flora*, indicating a close phylogenetic relationship. All *Dolichovespula* species analyzed formed a distinct monophyletic clade that was clearly separated from the genera *Vespa* and *Vespula*.

## Discussion and conclusion

In this study, we report the complete mitochondrial genome sequence of *D. kuami* for the first time. Phylogenetic analysis revealed that species of the genus *Dolichovespula* within the subfamily Vespinae form a well-supported monophyletic clade. The overall topology recovered in this study is similar to that reported by Wang et al. (2022), in that *Vespula* and *Vespa* were grouped together, with *Dolichovespula* placed as the most distantly related lineage within Vespinae; however, the *Vespula*–*Vespa* relationship received low bootstrap support (UFBoot = 47) in our analysis. The genus *Provespa*, which was not assessed in previous studies, clustered closely with *Vespa*, offering a more detailed view of genus-level relationships within Vespinae. Further research with broader taxon sampling and genome-scale datasets is crucial for resolving these relationships with greater confidence.

Within the *Dolichovespula* clade, *D. kuami* was recovered as a sister group to *D. flora*, indicating a close evolutionary relationship. However, the pairwise genetic distance between *D. kuami* and *D. flora* was calculated as 0.1196 ± 0.0032 substitutions per site (Kimura 2-parameter model, MEGA 12; Kumar et al. 2024), based on complete mitochondrial genome sequences. This level of divergence, along with their distinct mitochondrial lineages, supports the recognition of *D. kuami* as a valid and distinct species. These findings align with the taxonomic interpretation of Kim et al. (2024), who distinguished the two species based on morphological, ecological, and molecular characteristics.

These findings confirm the taxonomic status of *D. kuami* and establish a genomic basis for future studies on the taxonomy, phylogeny, and evolutionary history of Vespidae.

## Supplementary Material

FigS1(18cm,600).jpg

## Data Availability

The genome sequence data supporting this study’s findings are available in GenBank of NCBI at (https://www.ncbi.nlm.nih.gov) under assessment no. MK522808. The associated BioProject, Bio-Sample, and SRA numbers were PRJNA1304683, SAMN50565862, and SRR34954834. The RefSeq BioProjects corresponding to this sample is PRJNA927338.
