# Supplementary figures and images for: Complete mitochondrial genome sequence of Dolichovespula kuami (Hymenoptera: Vespidae)
Source: Mitochondrial DNA B Resour. 2026 Feb 1;11(3):340–4. doi: 10.1080/23802359.2026.2620190 (PMC12865839; doi:10.1080/23802359.2026.2620190)

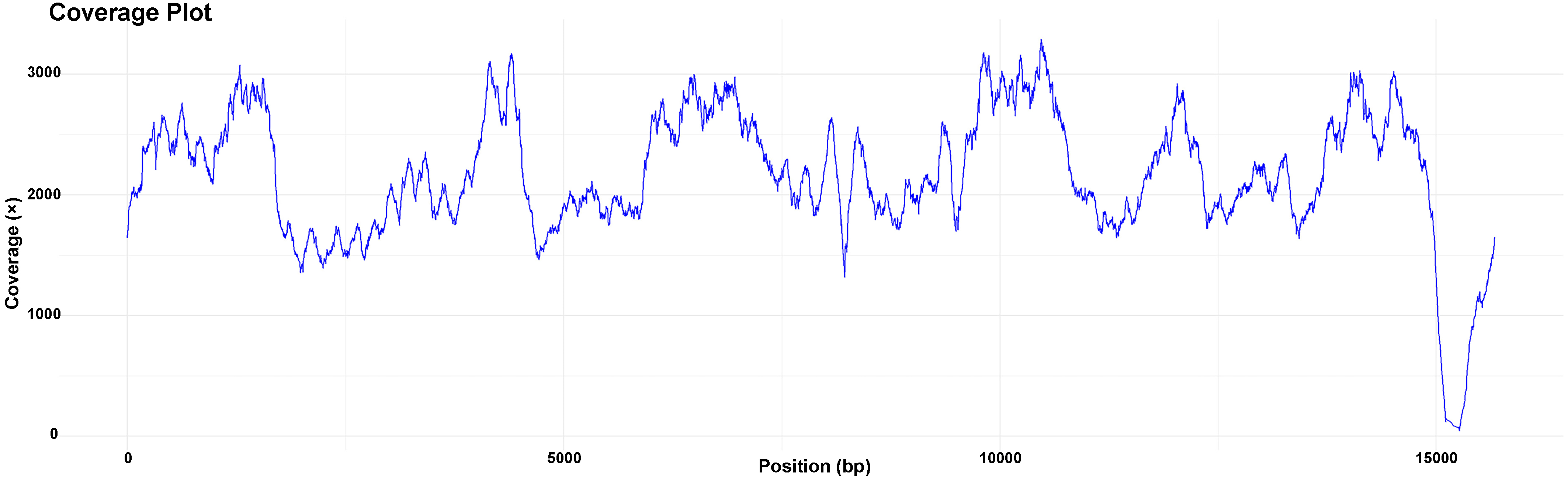

Supplement: FigS1(18cm,600).jpg [file TMDN_A_2620190_SM6324.jpg]
